# Effect of High Jugular Bulb on the Hearing Loss Characteristics in Patients With LVAS: A Pilot Study

**DOI:** 10.3389/fcell.2021.743463

**Published:** 2021-08-27

**Authors:** Arthur Benjamin Kwesi, Jintao Yu, Chenlu Wang, Yonghua Wang, Fengyi Chuang, Xiaohui Yan, Wendi Shi, Yu Sun

**Affiliations:** ^1^Department of Otorhinolaryngology, Union Hospital, Tongji Medical College, Huazhong University of Science and Technology, Wuhan, China; ^2^Hearing and Speech Rehabilitation Institute, Zhejiang Chinese Medical University, Hangzhou, China; ^3^Hangzhou Ren-ai Hearing Rehabilitation Research Centre, Hangzhou, China

**Keywords:** high jugular bulb, large vestibular aqueduct syndrome, hearing loss, hearing characteristics, pilot study

## Abstract

**Objective:**

Both large vestibular aqueduct syndrome (LVAS) and high jugular bulb (HJB) are regarded as abnormalities commonly seen on the temporal bone CT. High jugular bulb has been found to erode the vestibular aqueduct, and there are several studies on jugular bulb vestibular aqueduct dehiscence. However, there is no study that specifically reports LVAS with concurrent HJB and its hearing loss relatedness. This study presents the pure tone audiometry differences between LVAS with HJB, and LVAS without HJB.

**Methods:**

This was a case control study involving 36 bilateral LVAS with concurrent unilateral HJB patients, total of 72 ears. Intra-person comparison was done, by dividing ears into two groups: the case group, 36 ears (LVAS with HJB); and the control group, 36 ears (LVAS without HJB). Air conduction thresholds (250–4000 Hz), bone conduction thresholds (250–1000 Hz), and air bone gap (250–1000 Hz) were analyzed and compared between groups.

**Result:**

There were statistically significant differences in AC thresholds at 250, 500, 2000, and 4000 Hz between the groups, *p* < 0.05. But there was no statistical significant difference at 1000 Hz, *p* > 0.05. There were statistical significant differences in BC thresholds at 250 and 500 Hz, *p* < 0.05, but there was no statistical difference at 1000 Hz. There were no significant differences in air bone gap at 250, 500, and 1000 Hz between the two groups.

**Conclusion:**

LVAS with concurrent HJB was found to have higher air conduction thresholds, especially at 250, 500, 2000, and 4000 Hz. Bone conduction thresholds were higher at 250 and 500 Hz. Air bone gap at 250, 500, and 1000 Hz, were not significantly higher in LVAS with concurrent HJB.

## Introduction

Large vestibular aqueduct syndrome (LVAS) is defined as the enlargement of the narrow bony canals (aqueducts), which extends from the vestibule into the skull. It is known to cause about 5–15% of congenital sensorineural hearing loss in children (Madden et al., 2007; Kupfer et al., 2012; Noguchi et al., 2017). The vestibular aqueduct is determined as enlarged, by temporal bone CT showing the diameter of the aqueduct >1.5 mm at the midpoint or the operculum, or by MRI showing an enlargement of the endolymphatic duct and sac (Aimoni et al., 2017). Studies have revealed no direct relationship with the size of the enlarged vestibular aqueduct and the level of hearing loss (Yan et al., 2003; Zhang et al., 2006). High jugular bulb (HJB) is known as the elevation of the posterior part of the jugular bulb into any of the following parts in the temporal bone: the hypotympanum portion of the middle ear, the base of the cochlear base, and the internal auditory canal, either with dehiscence or without dehiscence (Tanrivermis Sayit et al., 2017). The cause of high jugular bulb could be linked to the imbalanced venous system, which drains blood from the head, and the effect of the blood flow dynamics (Friedmann et al., 2012). High jugular bulb is one of the most common anatomical variations found in the temporal bone and has been reported to have high occurrence in patients with inner ear anomalies (Singla et al., 2016; Tanrivermis Sayit et al., 2017). High jugular bulb has been found to erode inner ear structures including the vestibular aqueduct (Friedmann et al., 2012). Large vestibular aqueduct syndrome with concurrent high jugular bulb has been observed clinically in patients having hearing loss. However, there are no previous studies in literature about the large vestibular aqueduct syndrome with high jugular bulb and its hearing loss relatedness. Some patients with large vestibular aqueduct syndrome were observed to have worse degree of hearing loss, and easily experienced hearing fluctuation. Previous studies on high jugular bulb revealed worse hearing thresholds and progressive hearing in the affected ears of patients with bilateral hearing loss (Tsunoda, 2000; Sasindran et al., 2014). As a novel study, the purpose was to ascertain the audiological characteristics of vestibular aqueduct syndrome with concurrent high jugular bulb by finding out the differences in air conduction thresholds, bone conduction thresholds, and air bone gap between LVAS with HJB and LVAS without HJB. The findings of this study are intended to help ear and hearing health professionals to understand the differences that exist among LVAS patients, in order to guide their diagnosis, counseling, and hearing management, as well as to guide future research on LVAS and HJB.

## Materials and Methods

This is a case control study conducted at the Zhejiang Chinese Medical University’s Affiliated Hearing and Speech Rehabilitation Institute. The study protocol was approved by the Institutional Review Committee.

### Subject Recruitment

The study involved EVA patients who visited the hearing and speech rehabilitation center between June 2020 and March 2021 for follow-up. Subjects were recruited based on the following inclusion criteria: (1) diagnosed with bilateral large vestibular aqueduct syndrome with unilateral high jugular bulb using CT; (2) ability to perform behavioral audiometry; (3) stable hearing for at least a 3-month period; (4) patients having outer ear and middle ear abnormalities were excluded by otoscopy, CT, and tympanometry; and (5) an informed consent for participation in the study.

### Subjects

**FIGURE 1:**
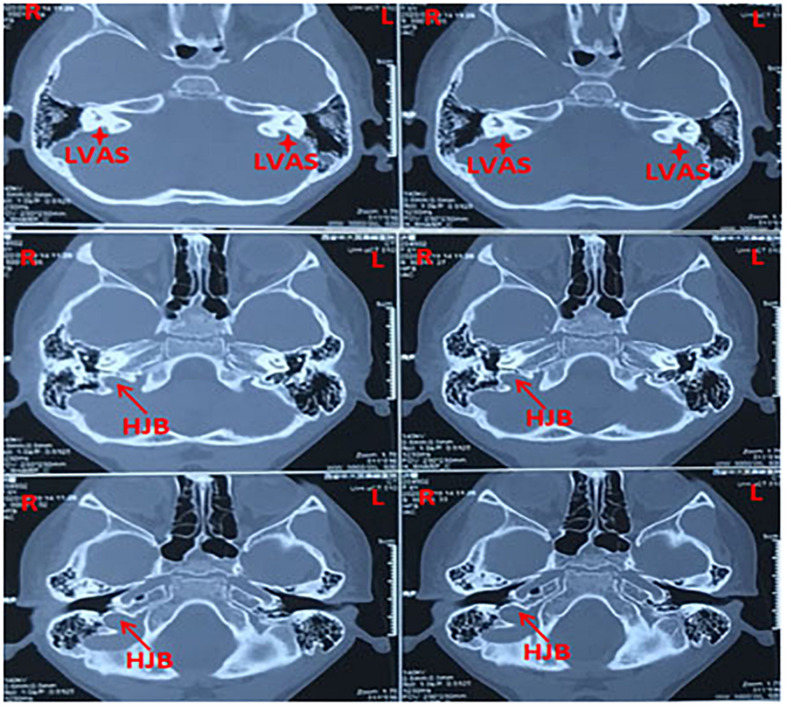
Representative CT (computed tomography) scan images showing bilateral large vestibular aqueduct syndrome with unilateral high jugular bulb (right ear) from a 3-years-old patient.

Thirty-six bilateral large vestibular aqueduct syndrome with unilateral high jugular bulb patients were recruited (Figure 1), comprising 19 males and 17 females; ages ranged from 3 to 27 years, and mean age was 8.08 years. A total of 72 large vestibular aqueduct syndrome ears, were divided into two groups: case group, 36 ears (LVAS with HJB) and control group, 36 ears (LVAS without HJB).

### Audiological Testing

All tests were performed in a clinical setting and in accordance with standard procedures. Pure tone audiometry and tympanometry were performed in consideration of the respective ages of patients.

#### Test Equipment

Pure tone audiometry was performed in a double-walled sound booth, using a Grason–Standler Audiostar ProTM audiometer, TDH-39, and B-71 transducers. Interacoustics Titan/IMP440 tympanometer was used to perform the tympanometry.

#### Pure Tone Audiometry

Using the Hughson Westlake threshold finding approach, air conduction and bone conduction thresholds were measured at octave frequencies from 250 to 4000 Hz. Masking was performed when necessary.

#### Tympanometry

Using 226 Hz probe tone at 85 dB SPL, and varying pressure from +200 to –400 daPa, the tympanic membrane mobility and middle ear compliance were assessed. Indications for normal tympanogram were ear canal volume, tympanometric peak pressure, and tympanometric peak compliance within the normative data for tympanometry for children and adults (Margolis and Heller, 1987).

### Variable and Comparison

Intra-person comparison was done between ears; 72 ears divided into the case group (36 ears) and the control group (36 ears). AC thresholds from 250 to 4000 Hz; BC thresholds from 250 to 1000 Hz; and air bone gap (ABG) from 250 to 1000 Hz were compared between the two groups.

### Statistical Analysis

Statistical analysis was conducted using the IBM SPSS version 25.0; paired-samples *t*-test was performed to determine the differences between the groups, *p* < 0.05.

## Results

### Demographic Characteristics and Radiological Findings

Thirty-six patients with bilateral large vestibular aqueduct syndrome with unilateral high jugular bulb constituted 19 males and 17 females, given a male to female ratio of 1.12:1; ages ranged from 3 to 27 years, and mean age was 8.08 years (Table 1).

**TABLE 1:** Gender and age characteristics of subjects.

Gender	<6 years	6–12 years	>12 years	Total
Male	4	10	5	19
Female	4	7	6	17

*NB: There was no statistical significance difference, *p* > 0.05.*

Seventy-two large vestibular aqueduct syndrome ears were divided in two groups: the case group, 36 ears (LVAS with HJB); and control group, 36 ears (LVAS without HJB). High jugular bulb presented in both left and right ears. The lateralization of high jugular was more in right ears as compared to the left ears. Thirty patients were found to have high jugular bulb in the right ear; 6 patients had high jugular bulb in the left ear, *p* < 0.05.

### Pure Tone Audiometry

#### Air Conduction Thresholds

The statistical analysis of air conduction thresholds from 250 to 4000 Hz showed the following mean thresholds for the case group: 250 Hz (61.94 ± 18.45 dB HL), 500 Hz (66.94 ± 19.50 dB HL), 1000 Hz (73.06 ± 19.10 dB HL), 2000 Hz (82.64 ± 20.96 dB HL), and 4000 Hz (89.44 ± 21.41 dB HL); for the control group: 250 Hz (54.31 ± 17.90 dB HL), 500 Hz (59.58 ± 17.94 dB HL), 1000 Hz (66.67 ± 18.60 dB HL), 2000 Hz (75.00 ± 16.48 dB HL), and 4000 Hz (78.47 ± 22.03 dB HL). The mean differences were 250 Hz (7.63 dB HL), 500 Hz (7.36 dB HL), 1000 Hz (6.39 dB HL), 2000 Hz (7.64 dB HL), and 4000 Hz (10.97 dB HL). Comparison between the two groups showed there were statistical significant differences in AC thresholds at 250, 500, 2000, and 4000 Hz. *p* Values were <0.05, but there was no statistical significant difference at 1000 Hz, *p* > 0.05 (Table 2). The tympanometry results also showed that there was no statistical significant difference in the tympanometric peak compliance between the case group (mean rank = 21.90), and the control group (mean rank = 26.52), *z* = −1.313, *p* = 0.189 > 0.05 (Supplementary Table 1).

**TABLE 2:** AC thresholds of case group and control group.

Frequency (Hz)	Case group (*n* = 36)	Control group (*n* = 36)
250	61.94 ± 18.45*	54.31 ± 17.90
500	66.94 ± 19.50*	59.58 ± 17.94
1000	73.06 ± 19.10	66.67 ± 18.60
2000	82.64 ± 20.96*	75.00 ± 16.48
4000	89.44 ± 21.41*	78.47 ± 22.03

Paired samples *t*-test *p < 0.05.

#### Bone Conduction Thresholds at Low and Mid Frequencies

The results showed the following mean thresholds for the case group: 250 Hz (20.69 ± 14.70 dB HL), 500 Hz (37.36 ± 15.42 dB HL), and 1000 Hz (51.11 ± 14.50 dB HL); for the control group: 250 Hz (15.56 ± 11.33 dB HL), 500 Hz (32.08 ± 13.91 dB HL), and 1000 Hz (48.75 ± 14.21 dB HL). The mean differences in bone conduction thresholds were 250 Hz (5.14 dB HL), 500 Hz (5.28 dB HL), and 1000 Hz (2.36 dB HL). Comparison between the two groups showed that there were statistically significant differences in BC thresholds at low frequencies: 250 and 500 Hz. *p* Values were: 250 Hz (*p* = 0.008 < 0.05), 500 Hz (*p* = 0.010 < 0.05), but there was no statistical significant difference at 1000 Hz, *p* = 0.245 > 0.05 (Table 3).

**TABLE 3:** BC thresholds of case group and control group.

Frequency (Hz)	Case group (*n* = 36)	Control group (*n* = 36)
250	20.69 ± 14.70*	15.56 ± 11.33
500	37.36 ± 15.42*	32.08 ± 13.91
1000	51.11 ± 14.50	48.75 ± 14.21

Paired samples *t*-test *p < 0.05.

#### Air Bone Gap at Low and Mid Frequencies

Statistical analysis of the air bone gap from 250 to 1000 Hz was performed in 36 patients, 72 ears (36 ears in the case group; 36 ears in the control group). The results showed the following mean ABG for the case group: 250 Hz (41.25 ± 14.46 dB HL), 500 Hz (30.00 ± 13.94 dB HL), and 1000 Hz (21.94 ± 13.11 dB HL); for the control group: 250 Hz (38.75 ± 15.23 dB HL), 500 Hz (27.50 ± 13.18 dB HL), and 1000 Hz (17.92 ± 11.91 dB HL). The mean differences in air bone gap were 250 Hz (2.50 dB HL), 500 Hz (2.50 dB HL), and 1000 Hz (4.03 dB HL). Comparison between the two groups showed there were no statistically significant differences in air bone gap at low and mid frequencies. *p* Values were 250 Hz (*p* = 0.379 > 0.05), 500 Hz (*p* = 0.320 > 0.05), and 1000 Hz (*p* = 0.078 > 0.05) (Table 4).

**TABLE 4:** Air bone gap of case group and control group.

Frequency (Hz)	Case group (*n* = 36)	Control group (*n* = 36)
250	41.25 ± 14.46	38.75 ± 15.23
500	30.00 ± 13.94	27.50 ± 13.18
1000	21.94 ± 13.11	17.92 ± 11.91

*Paired samples *t*-test *p* > 0.05.*

## Discussion

The laterality of high jugular bulb was found mostly in right ears, the study results showed 30 patients had high jugular bulb in the right ear, and six patients had high jugular bulb in the left ear. Some researches have reported the occurrence of high jugular bulb to be more in the right ears than left ears (Singla et al., 2016; Tanrivermis Sayit et al., 2017). The right side normally has the dominant venous system draining blood from the head, this blood flow dynamics could be linked to the cause of jugular bulb abnormalities. Right sided venous dominance was observed in majority of people with abnormal jugular bulb (Friedmann et al., 2012). The imbalanced system may be linked to the asynchronous development of the embryonic venous sinuses, which may lead to asymmetric blood flow during the early stages of cardiac venous pulsation (Wang et al., 2020). The prevalence of high jugular bulb in males and females and age categories are not significantly different, but the size or area of the jugular bulb may be different with males and females, and with different age categories (Friedmann et al., 2011; Wang et al., 2020).

### Air Conduction Thresholds of LVAS With HJB

The study findings revealed that AC thresholds of the case group were higher at all test frequencies, compared to the AC thresholds of the control group. There was a statistically significant difference between the AC thresholds of the two groups at 250, 500, 2000, and 4000 Hz, *p* values were <0.05; but there was no statistical significant difference at 1000 Hz, *p* > 0.05, although there was an increase of >6 dB HL. Previous studies on jugular bulb abnormalities found that hearing thresholds of the ears with high jugular bulb were higher compared to the ears without jugular bulb abnormalities (Tsunoda, 2000; Sasindran et al., 2014; Tanrivermis Sayit et al., 2017). There was an average increase of 8 dB HL in air conduction thresholds across all the tested frequencies when LVAS with HJB present in single ear.

#### Low-Frequency AC Thresholds of LVAS With HJB

Findings of this study revealed significant increase in AC thresholds at low frequencies (250 and 500 Hz), an average of >7 dB HL difference was found between the groups. High jugular bulb affects hearing in the low-frequency domain, by interfering with the conductive process hearing. HJB obstructs the tympanic membrane, and the movement of the ossicles, as well as impinges on the round window niche (Weiss et al., 1997; Chennupati et al., 2011; Barr and Singh, 2016; Toman et al., 2016). High jugular bulb presenting in the middle could reduce the middle ear’s efficacy to achieve proper impedance matching. In order for a proper impedance matching to be achieved, there needs to be an intact tympanic membrane, a normal ossicular chain, and a conducive middle ear space. There could be a possibility that high jugular bulb changes the normal physiology of the middle ear, hence making it difficult for the middle ear to effectively change the low-pressure high-displacement movements of the eardrum into high-pressure low-displacement movements, required for the cochlear fluid to move. When efficient impedance matching is not achieved, some low-frequency sound energy lost. The obstruction or closure of the round window has been found to cause a threshold shift, a phenomenon known as the cochlear conductive loss, which affects mostly the low-frequency sounds (Weiss et al., 1997). The third window leak out created by the enlarged vestibular aqueduct causes low-frequency sound energy to leak out of the cochlear (Arjmand and Webber, 2004; Merchant et al., 2007; Gopen et al., 2011). Therefore, the interference of the middle ear and obstruction of the round window membrane by HJB, and the third window leakage caused by LVAS, explains the increase in low-frequency thresholds when LVAS with HJB are present in one ear.

#### High-Frequency AC Thresholds of LVAS With HJB

The findings of our study revealed that there was a significant increase in AC thresholds at high frequencies, 2000 and 4000 Hz; there was an average difference of >9 dB HL between the groups. Enlarged vestibular aqueduct has been reported to cause endolymphatic reflux, which is the flow back of the endolymph into the cochlear. This causes electrolyte imbalance due to the high volumes of endolymph in the cochlear and affects the ionic pump mechanism of stria vascularis, which could lead to accumulation of toxic metabolites, which may damage the inner ear hair cells (Gopen et al., 2011; Chenlu et al., 2020). The high jugular bulb, which can be found impinging on the cochlear from the base, could add pressure onto the inner ear and disturb the inner ear’s homeostasis. The added pressure on cochlear, and electrolyte imbalance in the cochlear, affects the cochlear hair cells, which causes cochlear dead regions mostly at high frequencies. The study results showed 4000 Hz to have the highest increase in thresholds (10.97 dB HL), suggesting having LVAS with HJB in one ear, greatly affecting the hearing at high-frequency hearing thresholds.

There was no statistical significant difference at 1000 Hz, although there was an increase of >6 dB HL in thresholds. LVAS and HJB and the frequencies affect hearing in different frequency spectra. LVAS affects mostly high frequencies, which is seen by a steep sloping audiogram. The hearing loss of HJB is a rising configuration, meaning low frequencies are mostly affected. Now when LVAS and HJB occur in one ear, all frequencies may be affected, but mid-frequency standing waves could not be affected much. Also, the human ear is known to have higher sensitivity to sounds around 1000 Hz; this could explain the statistically insignificant difference between thresholds at 1000 Hz.

### Bone Thresholds of LVAS With HJB

The result showed bone conduction thresholds of the case group were higher than bone conduction thresholds of the control group. There were statistically significant differences in bone conduction thresholds at 250 and 500 Hz, *p* < 0.05, but there was no statistically significant difference at 1000 Hz, *p* > 0.05. Round window obstruction has been reported to affect bone conduction thresholds. Normally, vibrations of the osseous cochlear capsule would cause alternating compression and expansion of the labyrinthine fluids. The large volume of the scala vestibuli, relative to the scala tympani, sets a pressure differential across the two partitions of the cochlear duct, and produces a traveling wave in the direction of the scala tympani [16]. When the vibrations of the scala tympani are restricted by the closure of the round window, bone conduction would be affected due to compressed pressure retained in the cochlear. When there are no cochlear outlets, these compressed pressures would spill off forcefully through different outlets (third window), such as the cochlear aqueduct, vestibular aqueduct, semicircular canals, and vascular and neural channels (Weiss et al., 1997; Ho et al., 2017). In the case of LVAS with HJB in one ear, the enlarged vestibular aqueduct becomes the outlet for the compressed pressure to spill off and low-frequency vibrations to leak out. This explains the increased bone conduction thresholds found in LVAS with concurrent HJB. When the round window is immobile but there is an available acoustic outlet, low-frequency vibrations would escape efficiently with minimal bone conduction loss, and with increasing frequencies, the efficiency of the leakage decreases due to a rising impedance and bone thresholds become higher (Weiss et al., 1997). This supports the reason for which the result showed a significantly higher BC threshold at low frequencies, but was not significantly higher at 1000 Hz.

### Air Bone Gap of LVAS With HJB

The study results showed there was no statistical significant difference in air bone gap between the two groups, *p* > 005. The mechanism of air bone gap in LVAS patients is caused by the third window created by the enlarged vestibular aqueduct and dehiscence of the semicircular canals. This abnormal opening in the bony labyrinth changes the compliance of the inner ear and results in sound energy being leaked out of the cochlea (Gopen et al., 2011). LVAS increases the impedance difference between scala vestibuli and scala tympani of the cochlear partition by lowering impedance on the vestibuli side, thereby improving cochlear response to bone conduction (Merchant et al., 2007). However, in the case of large vestibular aqueduct syndrome with concurrent high jugular bulb, the mechanism changes. Although LVAS lowers impedance on the vestibuli side to improve the cochlear response to sound vibrations, the obstructed round window membrane would still restrict the formation of the sound traveling wave pressure in the inner ear. This causes the bone conduction thresholds to fall on the audiogram. A study on high jugular bulb reported that both air conduction and bone conduction thresholds would fall simultaneously, by obstruction of the round window membrane with an available leak outlet and the interference with the traveling wave by an additional inner ear pressure (Tsunoda, 2000). This supports our study findings showing increase in both air conduction and bone conduction thresholds in LVAS with HJB. Air bone gap could still be seen on the audiogram of LVAS with HJB, but due to the synchronous decline on air conduction and bone conduction thresholds, the difference would not be high, when compared with LVAS without HJB.

The study findings predict that when LVAS and HJB are together in one ear, it affects low-frequency threshold by the conductive component, caused by the obstruction of the eardrum and ossicles, blocking of the round window niche, and the third window. High = frequency thresholds are affected by increased inner ear pressure and cochlear dead regions. This may account for the significant increase in air conduction thresholds at 250, 500, 2000, and 4000, as well as the increase in bone conduction thresholds at 250 and 500 Hz. Nevertheless, these findings may not be representative of all LVAS with HJB patients, since during the process of this research, some LVAS with HJB still had better thresholds than LVAS without HJB. Therefore, there may be other factors that could contribute to threshold increase in patients with LVAS, but this study presents HJB as a factor that could contribute to higher hearing thresholds in LVAS patients.

## Conclusion

LVAS with HJB would have higher air conduction thresholds, especially at 250, 500, 2000, and 4000 Hz. Bone conduction thresholds would also be higher at 250 and 500 Hz. LVAS with HJB would have air bone gap at 250, 500, and 1000 Hz, but air bone gap would not be significantly higher than LVAS with HJB.

## Data Availability Statement

The raw data supporting the conclusions of this article will be made available by the authors, without undue reservation.

## Ethics Statement

The studies involving human participants were reviewed and approved by Zhejiang Chinese Medical University’s Affiliated Hearing and Speech Rehabilitation Institute. The patients/participants provided their written informed consent to participate in this study. Written informed consent was obtained from the individual(s) for the publication of any potentially identifiable images or data included in this article.

## Author Contributions

YW, WS, and YS contributed to the conception and design of the study and revised the manuscript. AK, CW, FC, and XY collected the data and performed the statistical analysis. AK and JY drafted and revised the manuscript. All authors have read and approved the submitted version.

## Conflict of Interest

The authors declare that the research was conducted in the absence of any commercial or financial relationships that could be construed as a potential conflict of interest.

## Publisher’s Note

All claims expressed in this article are solely those of the authors and do not necessarily represent those of their affiliated organizations, or those of the publisher, the editors and the reviewers. Any product that may be evaluated in this article, or claim that may be made by its manufacturer, is not guaranteed or endorsed by the publisher.
