# Supplementary material for: Effect of High Jugular Bulb on the Hearing Loss Characteristics in Patients With LVAS: A Pilot Study
Source: Front Cell Dev Biol. 2021 Aug 27;9:743463. doi: 10.3389/fcell.2021.743463 (PMC8429953; doi:10.3389/fcell.2021.743463)
Supplement: Supplementary file 1 [file Table_1.doc]

| **Supplementary** **table 1.** Tympanometric peak compliance of case and control group | | | | |
| --- | --- | --- | --- | --- |
|  | Median | IQR | z | p |
| Case group | 0.41 | 0.18 |  |  |
|  |  |  | -1.31 | 0.189 |
| Control group | 0.42 | 0.27 |  |  |
| Wilcoxon signed-ranks test p>0.05 | | | | |
